# The Unresolved Methodological Challenge of Detecting Neuroplastic Changes in Astronauts

**DOI:** 10.3390/life13020500

**Published:** 2023-02-11

**Authors:** Ford Burles, Rebecca Williams, Lila Berger, G. Bruce Pike, Catherine Lebel, Giuseppe Iaria

**Affiliations:** 1 Canadian Space Health Research Network, Department of Psychology, Hotchkiss Brain Institute, Alberta Children’s Hospital Research Institute, University of Calgary, Calgary, AB T2N 1N4, Canada; 2 Faculty of Health, School of Human Services, Charles Darwin University, Darwin, NT 0810, Australia; 3 Department of Radiology, Department of Clinical Neuroscience, Hotchkiss Brain Institute, University of Calgary, Calgary, AB T2N 1N4, Canada; 4 Department of Radiology, Alberta Children’s Hospital Research Institute, Hotchkiss Brain Institute, University of Calgary, Calgary, AB T2N 1N4, Canada

**Keywords:** space medicine, brain volumetry, microgravity

## Abstract

After completing a spaceflight, astronauts display a salient upward shift in the position of the brain within the skull, accompanied by a redistribution of cerebrospinal fluid. Magnetic resonance imaging studies have also reported local changes in brain volume following a spaceflight, which have been cautiously interpreted as a neuroplastic response to spaceflight. Here, we provide evidence that the grey matter volume changes seen in astronauts following spaceflight are contaminated by preprocessing errors exacerbated by the upwards shift of the brain within the skull. While it is expected that an astronaut’s brain undergoes some neuroplastic adaptations during spaceflight, our findings suggest that the brain volume changes detected using standard processing pipelines for neuroimaging analyses could be contaminated by errors in identifying different tissue types (i.e., tissue segmentation). These errors may undermine the interpretation of such analyses as direct evidence of neuroplastic adaptation, and novel or alternate preprocessing or experimental paradigms are needed in order to resolve this important issue in space health research.

## 1. Introduction

With NASA’s Artemis plan [1] including crewed space missions to the Moon and prospective missions to Mars, there is a significant interest in understanding the health risks associated with long-duration spaceflight [2] and, in particular, its effects on the behaviour, cognitive performance, and neurological functioning of astronauts [3]. Such concerns are raised due to the unique and hazardous environment that astronauts are exposed to during spaceflight, with many known factors that can alter or impair their neurological functioning, such as weightlessness, radiation, isolation, and confinement [4].

One of the earliest, and now commonly-reported, neurological effects of spaceflight is a salient redistribution of cerebrospinal fluid (CSF) within the astronaut’s skull [5,6,7,8,9,10,11,12]; that is, the brain largely ‘floats upward’ and displaces fluid from the topmost portions of the intracranial cavity to its bottommost portions and the ventricles. In a clinical setting, such as the study of neurodegenerative conditions such as Alzheimer’s disease, an increase in ventricular volumes is indicative of increased global CSF and, therefore, a loss of brain tissue. However, most studies [13] investigating the effects of spaceflight in astronauts find that their total CSF volume does not change, nor does their overall brain tissue volume [5,11,14,15], indicating that spaceflight is not grossly neurodegenerative. Nevertheless, many studies have identified some local changes in the volume of specific brain regions in astronauts following a spaceflight. These include decreased grey matter volume in the frontal, temporal, and occipital lobes [9,11,14], with marginally increased volumes in the postcentral [14] and supplementary motor cortex [6]; some opposite findings [7] reported grey matter volume increases in the temporal and frontal cortex, with no focal losses. Altogether, these findings seem to indicate that spaceflight is, indeed, producing local volumetric changes in the brain of astronauts.

The neurological effects of spaceflight are typically construed [16,17] to largely arise from either (1) a direct physical impact of the typical spaceflight environment on the brain, or (2) an indirect neuroplastic change in response to the astronauts’ experience with microgravity and adaptation to the spaceflight environment. The first, ‘direct physical’, cause is best characterized by the mechanical forces exerted on the brain by the change in gravitational force, which produces a headward shift of fluid in the body, as well as causes the brain to shift upwards and creates local changes in CSF distribution; these changes may accumulate over time while astronauts are in space [12,15], but are ostensibly caused directly by exposure to microgravity. The second, ‘indirect’, cause attributed to neurological changes due to spaceflight are the neuroplastic changes that result from astronauts’ functional adaptation to spaceflight [4,16], i.e., the brain responding to behavioural and cognitive changes made by astronauts while they adjust to their new environment.

These two categories, as causal mechanisms, are neither exhaustive nor mutually exclusive. However, the neurological effects generally attributable to direct exposure to the spaceflight environment currently have little interpretive value from a cognitive neuroscience perspective. For instance, CSF is not a functional tissue, and microgravity-induced changes in local CSF volume are not expected to be directly associated with changes in cognition or behaviour. That is not to say that CSF does not play an important role in the brain, as CSF is known to provide structural support in the forum of buoyancy, and functional support in the form of waste removal and nutrient delivery, among other functions [18,19] and undoubtedly interacts with neighbouring neuronal tissue. However, changes in local CSF volume are not directly indicative of neuroplastic adaptation or degradation in the same way that local changes in grey matter volume can be [20,21]. Neurological adaptations to spaceflight, including those linked to changes in grey matter volume, provide insight into the behavioural and cognitive challenges faced by astronauts, as well as the mechanisms underlying their adaptations to this new environment [16,22,23]. These neuroplastic changes are particularly interesting as they may not always manifest in directly observable behaviours; therefore, understanding these neuroplastic effects could reveal unique insights and avenues for countermeasures or augmentations to improve astronauts’ performance, safety, and quality of life while on long-duration spaceflights. The disparity in interpretive value of these effects underscores the importance of dissociating the direct effects of spaceflight (e.g., fluid redistribution) from the neuroplastic responses to a spaceflight environment.

Investigating volumetric changes in the human brain through magnetic resonance imaging (MRI) requires a series of important data processing steps [24]. One critical preprocessing step before performing many volumetric analyses is to classify each voxel of a brain image among a handful of tissue types, a procedure called ’tissue segmentation’. These tissue types, which are typically differentiated on their signal intensities and spatial locations [25], can be neuronal tissues with functional relevance (e.g., grey and white matter) or other tissues with far lesser direct functional relevance such as skull and skin. Unfortunately, in certain parts of the brain, the dura (the connective tissue that encapsulates the brain) is directly adjacent to cortical grey matter and has a very similar intensity on typical structural MRI images, making it difficult to distinguish where the grey matter ends and the dura begins. As such, the automated segmentation of anatomical MRI images has a well-known tendency to incorrectly classify some non-neuronal tissues, such as dura, as grey matter [26,27]. The aforementioned CSF shift observed in astronauts between preflight and postflight timepoints [10] results in the dura being closer to the top of the brain and more distant from the bottom. This change in separation between the brain and dura alters the location at which these segmentation errors are most likely to occur. Therefore, the change in the spatial bias of segmentation errors could then manifest in apparent tissue ‘losses’ or ‘gains’—which may be merely ‘losses’ or ‘gains’ in misclassified tissue [28,29,30]. This is particularly troublesome, as changes in grey matter volume are typically indicative of neuroplastic processes, but this common segmentation error could make this interpretation not valid in studies of spaceflight.

In this study, we analyzed pre- and post-spaceflight MRI data from 43 astronauts to test the hypothesis that the grey matter segmentation errors produced during preprocessing of MRI data are exacerbated by the upward astronauts’ brain shift (and downward CSF redistribution) resulting from the extended exposure to microgravity during spaceflight. Our findings provide the very first evidence that observable grey matter volume changes—which would typically be interpreted as neuroplastic adaptations to spaceflight [4,16]—appear to be driven by segmentation errors resulting from the microgravity-related CSF redistribution and tissue displacement. First, we demonstrate that the spaceflight-related fluid redistributions produce a common pattern of errors in classifying different types of tissue in the brain; then, we show that these errors manifest as artifactual grey matter gains and losses and are not indicative of actual neuroplastic responses to spaceflight. We further illustrate that ‘multimodal segmentation’—a procedure known to be more robust to these types of segmentation errors [26,31,32]—is unable to satisfactorily resolve these misclassifications. The ubiquity of these errors suggests that novel data acquisition or preprocessing pipelines will need to be identified and developed to permit valid neuroplastic interpretations of any spaceflight-related changes in local brain volume.

## 2. Materials and Methods

### 2.1. Participants

We analyzed MRI data from 32 astronauts from the NASA Lifetime Surveillance of Astronaut Health Program, as well as 11 astronauts who participated in the Canadian Space Agency (CSA) project ‘Wayfinding’. This study was approved by the institutional review boards of NASA’s Johnson Space Center and the University of Calgary. All participants provided written informed consent, and NASA has reviewed this manuscript and ensured it is compliant with the privacy standards of the NASA Astronaut Office. Our sample was composed of 10 female and 33 male participants (Mean age = 47.79, SD = 5.06 years at launch). Seven participants underwent short ~14-day spaceflights and the remainder were on multi-month spaceflights (Mean mission duration = 158.27, SD = 79.08 days). All participants underwent MRI scans pre- (Mean days before launch = 381.69, SD = 213.66) and post- flight (Mean days after return = 6.68, SD = 5.79).

### 2.2. MRI Data Acquisition

MRI data for all participants were acquired on a 3T Siemens Verio at the League City University of Texas Medical Branch Campus using a 32-channel head coil. Analyzed retrospective data from the NASA Lifetime Surveillance of Astronaut Health Program included a T1-weighted MPRAGE sequence (TR = 1.9 s, TE = 2.32 ms, TI = 0.9 s, FA = 9°, sagittal acquisition with in-plane resolution of 0.4883 by 0.4883 mm, and a slice thickness of 0.9 mm), and a T2-weighted sequence (TR = 3.2 s, TE = 409 ms, FA = 120°, sagittal acquisition with in-plane resolution of 0.4883 by 0.4883 mm, and a slice thickness of 1 mm), collected on Syngo versions B17 and B19. Analyzed data from the CSA ‘Wayfinding’ project included a T1-weighted MPRAGE sequence (TR = 2.3 s, TE = 2.34 ms, TI = 0.9 s, FA = 8°, sagittal acquisition with in-plane resolution of 0.9766 by 0.9766 mm, and a slice thickness of 1 mm) and a FLAIR sequence (TR = 5 s, TE = 354 ms, TI = 1.8 s, FA = 120°, sagittal acquisition with in-plane resolution of 0.9766 by 0.9766 mm, and a slice thickness of 1 mm), collected on Syngo version B19.

### 2.3. MRI Data Preprocessing

We first reoriented the T2-weighted images (i.e., T2 and FLAIR) to a standard orientation using fslreorient2std, included in FSL 6.0.4 [33]. Then, we used SPM12 v7771 [34] to perform a rigid-body, normalized mutual information coregistration of each subject-timepoint’s T2-weighted image to the corresponding T1-weighted image, and resampled the T2-weighted image to match the T1-weighted image’s voxel dimensions. For the ‘unimodal’ segmentation protocol, the T1-weighted images were segmented using SPM12′s default unified segmentation procedure, except using 2 Gaussians each for grey and white matter tissue classes [35], and a 1 mm separation distance. For the ‘multimodal’ segmentation protocol, we included the resampled T2-weighted image as an additional channel, with all other parameters held constant from the ‘unimodal’ segmentation protocol. SPM’s default segmentation procedure includes intensity normalization preceding the segmentation steps. For all subsequent preprocessing steps, we processed the unimodal and multimodal streams independently. To warp the subjects’ data to MNI space, we then created DARTEL [36] templates from the dartel-imported grey matter, white matter, and CSF tissue classes and, subsequently, DARTEL-normalized these images to MNI space at 1.5 mm isotropic resolution, preserving tissue amounts (i.e., ‘modulated’) and, subsequently, smoothed these modulated tissue maps with an 8 mm FWHM Gaussian kernel. Then we subtracted each smoothed, modulated, normalized preflight tissue map from their corresponding postflight tissue map to produce tissue volume change maps for analysis.

### 2.4. Voxel-Based Morphometry Analysis

Independently for the unimodal and multimodal segmentation preprocessing streams, we entered each subject’s grey matter tissue change maps into one-sample t-test analyses in SPM12, with the participants’ sex, age at launch, spaceflight duration, post-landing MRI delay, and study group (NASA retrospective and ‘Wayfinding’ studies) modelled as mean-centered covariates. We expect that the ‘study group’ covariate would account for systematic effects of the different MRI acquisitions utilized in each group. We restricted the analyses using implicit masking—ignoring voxels with no data—as well as an explicit mask which constrained the analysis to voxels with values exceeding 0.1 in SPM12s default grey matter tissue probability map, and excluded the brainstem, as defined in the ‘MNI structural atlas’ included in FSL. The brainstem was excluded from our analyses because it has poor contrast between grey and white matter, varying iron content, and is particularly sensitive to physiological noise [37,38]. No global normalization was applied. We thresholded the analyses using a two-tailed voxel height threshold of *p_FWE_* < 0.05, and only reported clusters with extents equal to or exceeding 40 voxels (i.e., 135 mm^3^). All coordinates are reported in MNI space.

### 2.5. Manual Segmentation

To provide a ‘ground truth’ state for comparison, we segmented the grey matter within two regions of interest from ten subjects each. Regions of interest were selected to encompass the largest grey matter ‘gain’ and ‘loss’ detected in the unimodal voxel-based morphometry analysis, i.e., clusters 1 and 2 identified in Table A1. The first ROI ‘tentorium’ encompassed the medial occipital cortex and cerebellar tentorium, spanning a rectangular prism from MNI −25, −99, −22 to MNI 25, −44, 18. The second ROI ‘falx’ encompassed the medial precentral gyrus and cerebral falx, spanning a rectangular prism from MNI −20, −51, 37 to MNI 20, −1, 87. For each ROI independently, we selected subjects for tracing by first rank-ordering subjects based on the effect size at the peak voxel identified in Table A1; i.e., MNI −2, −74, −6 for the ‘tentorium’ ROI, and MNI 6, −16, 63 for the ‘falx’ ROI. Then, from our 43 subjects, we selected the subjects with the 4th, 8th, …, 36th and 40th largest effects at each ROI to ensure a uniform sampling of effects across the study. Files for tracing were prepared by taking MNI-normalized T1 images restricted to the aforementioned coordinates, at a 0.8 mm isotropic resolution to improve the fidelity of the manual segmentations. The two raters, F.B. and L.B., first traced the grey matter from a pair of test ROIs, and a sufficiently high inter-rater agreement was met (mean Dice similarity of 0.80). Then, L.B. traced all ‘tentorium’ ROIs and F.B. traced all ‘falx’ ROIs. Raters were blind to the automated segmentation delineations and effect sizes while tracing. Utilizing the deformation fields calculated in the voxel-based morphometry analysis, manual tracings were warped to 1mm isotropic native space for quantification.

### 2.6. Comparison to Other Segmentation Algorithms

All subjects selected for manual segmentation were additionally processed by utilizing segmentation implementations from Advanced Normalization Tools (ANTs) ‘ATROPOS’ (v2.3.5) [39], CAT12 (r1860) [40], FSL ‘FAST’ (v6.0.4; fsl.fmrib.ox.ac.uk, accessed on 4 February 2023) and Freesurfer (v7.1.1; surfer.nmr.mgh.harvard.edu, accessed on 4 February 2023). Generally, default settings were selected for each algorithm, except where noted. These settings are intended to be representative of typical, as opposed to ideal, performance from these pipelines. Many software packages do not provide clear documentation on best practices for multimodal segmentation, so poor performance from multimodal segmentation should be interpreted with caution and is not indicative of any shortcomings of that particular software’s general capacity to perform accurate segmentation.

#### 2.6.1. Advanced Normalization Tools Pipeline

Subjects’ T1 and T2-weighted images were first bias-field corrected using ‘N4BiasFieldCorrection’. For the unimodal pipeline, the ‘antsBrainExtraction.sh’ script was used to extract the brain from the bias-field corrected T1 image, and the brain-extracted image was segmented using ‘Atropos’ with a k-means initialization with k = 3. The multimodal pipeline differed from the unimodal pipeline in that the bias-field removed T2 images were included as an additional anatomical image in the ‘antsBrainExtraction.sh’ script, and subsequently as an additional intensity image in ‘Atropos’. Multimodal segmentation failed (i.e., segmentations were extremely poor quality) in 3 subjects’ preflight images, and these were excluded from subsequent analysis. Segmentations produced by ‘Atropos’ were resampled to match the native-space manual segmentation space using SPM.

#### 2.6.2. CAT12 Pipeline

Subjects’ T1 images were entered into CAT12′s ‘Segment’ pipeline utilizing default parameters, and native-space grey matter segmentations selected as an output. The resultant grey-matter maps were then resampled to match the native-space manual segmentation space using SPM. Independently, the T1 images were processed in CAT12′s ‘Longitudinal Segment’ pipeline, utilizing default parameters. Grey matter segmentations from the longitudinal pipeline were resampled to match the native-space manual segmentation space using SPM. CAT12 does not support multimodal segmentation.

#### 2.6.3. Freesurfer Pipeline

Subjects’ T1 images were processed through Freesurfer’s recon-all command, including autorecon1, 2, and 3. Multimodal segmentation was performed by including the additional channel as an input in the recon-all command, as well as including flags to adjust the pial surface (e.g., -T2pial). For both unimodal and multimodal pipelines, the left hemisphere and right hemisphere grey matter mask (i.e., the volumetric cortical ribbon file) were merged and resampled to match the native-space manual segmentation space using SPM.

#### 2.6.4. FSL Pipeline

Subjects’ T1 images were denoised using FSL’s SUSAN with a 3 × 3 × 3 voxel SD mask. These images were subsequently brain-extracted using BET with the -B flag to reduce input image bias and remove residual neck voxels. The brain-extracted images were then entered info FAST, and the grey matter partial volume outputs were resampled to match the native-space manual segmentation space using SPM. Visual inspection revealed multimodal BET and FAST segmentation performance utilizing default parameters was poor, so these data were not included.

#### 2.6.5. Manual-to-Automated Comparisons

For each ROI, we computed the spaceflight-related percent change in grey matter volume using the following formula:

$$\frac{PostflightGMVolume-PreflightGMVolume}{PreflightGMVolume}$$

We utilized one-tailed paired-samples *t*-tests (α < 0.05) to compare the percent change computed by each automated algorithm with that computed from manual segmentation. Additionally, we performed one-sample *t*-tests (α < 0.05) to identify if any given segmentation method detected non-zero grey matter changes.

## 3. Results

### 3.1. Voxel-Based Morphometry Analysis: Unimodal Segmentation

**Figure 1:**
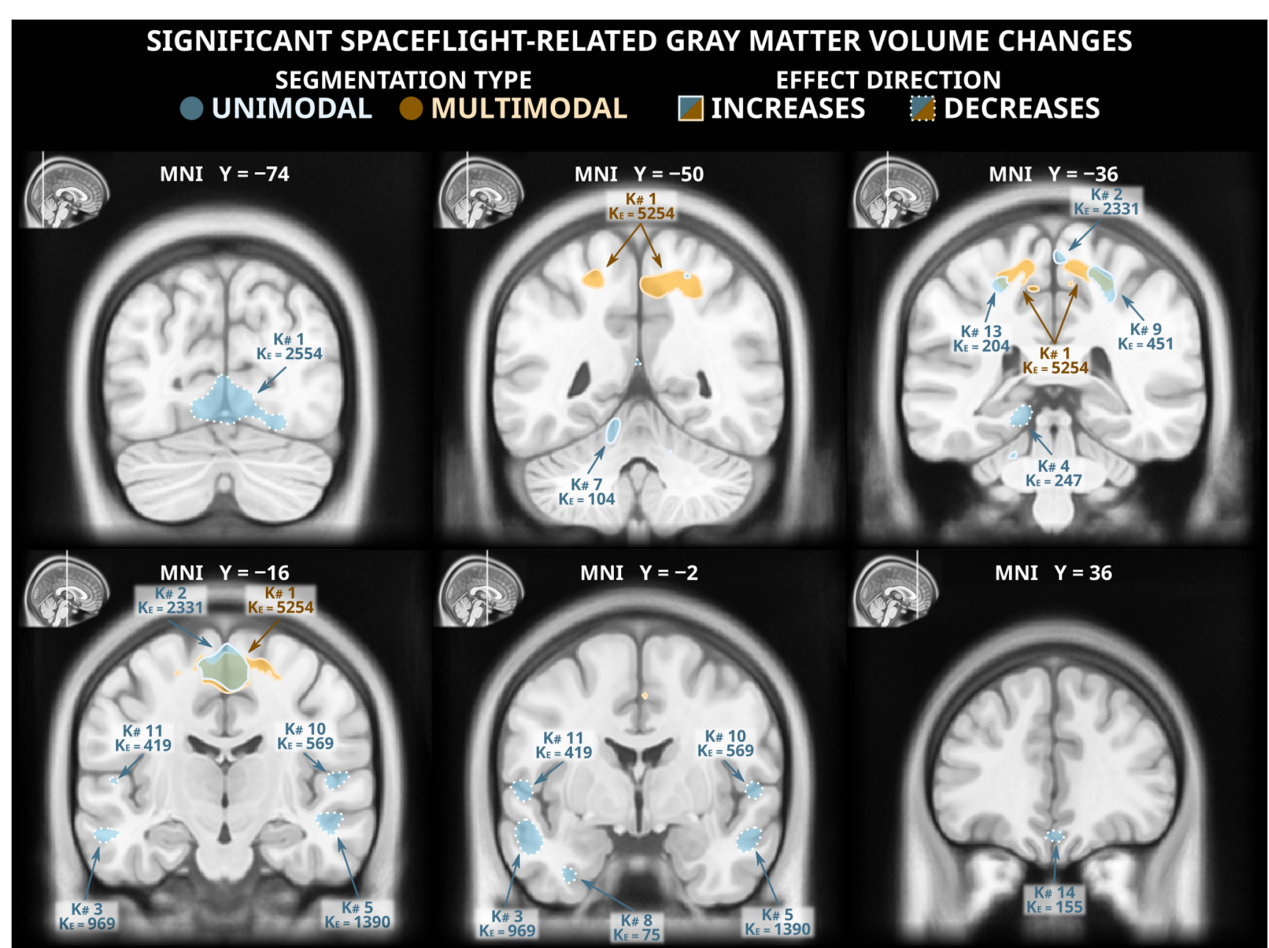
Statistically significant (two-tailed *p_FWE_* < 0.05) grey matter volume changes detected in a voxel-based morphometry analysis following unimodal (blue) and multimodal (orange) segmentation. Both analyses identified grey matter gains (solid outlines) in dorsomedial frontal and parietal cortex, and the unimodal analysis detected numerous clusters of significant grey matter losses (dashed outlines) in multiple locations across the occipital, temporal, and frontal lobes. The MNI ICBM 2009b Nonlinear Asymmetric template brain was used as a background. K#s reference the cluster numbers in the tabled results in Table A1 and Table A2 for unimodal and multimodal clusters, respectively.

The voxel-based morphometry analysis following unimodal segmentation detected widespread volumetric changes associated with spaceflight (Figure 1 and Table A1), with a total of 10,056 voxels reaching statistical significance (two-tailed *p_FWE_* < 0.05). The detected grey matter losses (totalling 6758 significant voxels) included clusters in the ventromedial occipital lobe (including the lingual gyrus), the lateral temporal cortex, the ventral temporal cortex, the medial temporal cortex (including the posterior parahippocampal cortex), the ventromedial frontal cortex, and the ventral cerebellum. The unimodal analysis detected less extensive grey matter gains (totalling 3298 significant voxels) including clusters in the paracentral lobule and the postcentral sulcus, the precuneus, and the cerebellum.

### 3.2. Voxel-Based Morphometry Analysis: Multimodal Segmentation

Of the statistically significant voxels detected in the unimodally-segmented analysis, only 2213 (~22%) were also flagged as significant in the multimodally-segmented analysis, which detected a total of 5559 voxels with statistically significant grey matter volume changes (Figure 1 and Table A2). These changes were all grey matter gains; no significant grey matter losses were detected in the analysis following multimodal segmentation. The detected grey matter gains were largely extensions and combinations of the grey matter gains detected following unimodal segmentation. This analysis detected a large cluster spanning the precuneus, the paracentral lobule, and the supplementary motor area as well as smaller clusters in the central sulcus and the supplementary motor area.

### 3.3. Artifacts Unique to Unimodal Segmentation

**Figure 2:**
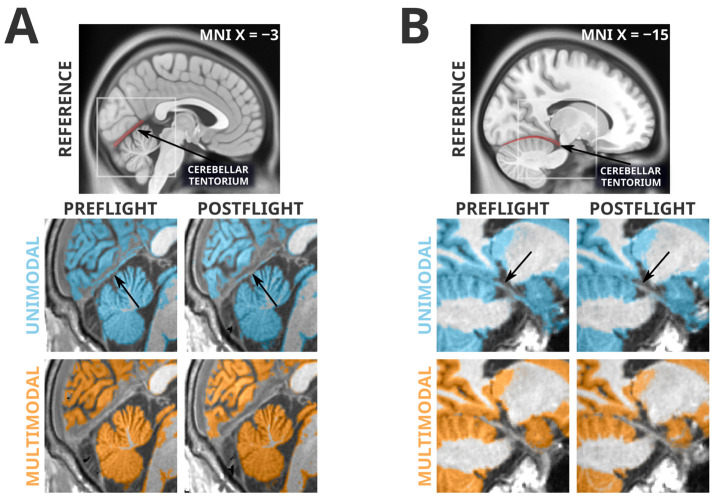
Spatially normalized grey matter segmentation exemplars illustrating the errors in unimodal segmentation that multimodal segmentation appears to attenuate. Highlighted regions were classified as grey matter in SPM12’s unified segmentation in unimodal (blue) and multimodal (orange) paradigms. Arrows indicate locations of interest in which differences in segmentation errors between preflight and postflight are visible. Panel (**A**) depicts a typical error (likely driving unimodal cluster K1) in which the cerebellar tentorium is classified as grey matter in the unimodal segmentation. In the preflight timepoint, because the grey matter of the ventral occipital and temporal cortex is resting upon the tentorium, this is often classified as grey matter. At the postflight timepoint there is a larger gap between the tentorium and the cerebral grey matter, decreasing the likelihood it is classified as grey matter, and producing an artifactual ‘loss’ of grey matter. The multimodal segmentation is less likely to classify the tentorium as grey matter, therefore making it less sensitive to this artifact. Panel (**B**) depicts a similar artifactual pattern in a different subject (this type of error likely driving unimodal cluster K4) in the temporal lobe, near the posterior hippocampus. Again, the unimodal segmentation has a tendency to classify a significant portion of the tentorium as grey matter at preflight timepoints, and then ‘loses’ some of this ‘grey matter’ at postflight when there is slightly more CSF separating the cortex from the tentorium.

The failure of the multimodal segmentation paradigm to detect the grey matter losses identified in the unimodal segmentation paradigm suggests that these grey matter losses may be artifactual. Figure 2 depicts examples of the types of unimodal segmentation errors we believe are driving these artifactual findings. Most saliently, these errors involve misclassification of the cerebellar tentorium, the dural structure separating the cerebellum from the ventral portion of the occipital and temporal lobes. The tentorium provides structural support to the cerebrum, preventing it from sagging under the effects of gravity and protecting the cerebellum underneath [41]. At preflight timepoints, the ventral cerebral grey matter is pressed against the tentorium, and in unimodal segmentation paradigms the tentorium is quite regularly incorrectly classified as grey matter. However, at postflight timepoints, there is slightly more CSF separating the tentorium from the nearby cortex, causing a smaller amount of the tentorium to be incorrectly classified as grey matter, and producing an erroneous ‘loss’ of grey matter volume. The multimodal segmentation paradigm is not as susceptible to this error, as it is less likely to erroneously classify the tentorium as grey matter at preflight or postflight timepoints.

### 3.4. Artifacts Present in Unimodal and Multimodal Segmentation

**Figure 3:**
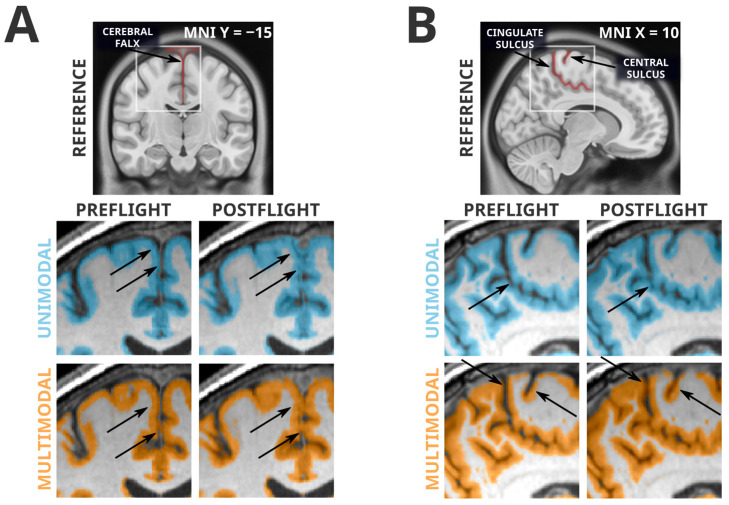
Spatially normalized grey matter segmentation exemplars illustrating errors conserved across unimodal and multimodal segmentation. Highlighted regions were classified as grey matter in SPM12’s unified segmentation in unimodal (blue) and multimodal (orange) paradigms. Arrows indicate locations of interest in which differences in segmentation errors between preflight and postflight are visible. Panel (**A**) depicts a classification error of the cerebral falx from a single subject. Both unimodal and multimodal segmentations appear to classify a greater portion of this dural structure as grey matter at postflight timepoints, likely because the CSF space that separates the cortex from the cerebral falx is much smaller at postflight. Similarly, Panel (**B**) depicts segmentations from a different subject, illustrating the tendency for the narrowed CSF spaces at postflight to be misclassified as grey matter, possibly producing artifactual tissue gains that are not indicative of neuroplastic effects. In addition, note the difficulty for automated segmentation procedures to correctly classify the highly-myelinated grey matter of the primary sensory and motor areas [42] as seen in Panel (**B**).

Whereas all grey matter losses detected in the unimodal segmentation paradigm were absent in the more reliable multimodal segmentation paradigm, many of the grey matter volume gains were conserved between the two analyses. However, this mere fact alone is not evidence of a lack of artifactual influence. From visual inspection, the multimodal segmentation generally appears to have fewer and less severe segmentation errors, but there remain clear artifacts contributing to the grey matter volume increases seen in both analyses. Examples of these artifacts are shown in Figure 3. Very similarly to the artifactual losses involving the cerebellar tentorium, the unimodal and multimodal segmentation paradigms were both susceptible to misclassification of the cerebral falx. The cerebral falx is a dural structure running in and along the longitudinal fissure separating the left and right hemispheres of the brain. At postflight timepoints, where the cortex is distanced from the tentorium at the ventral portions of the cerebrum, it crowds the cerebral falx at dorsal and midline portions of the cerebrum. Both segmentation paradigms had a tendency to incorrectly flag grey matter volume gains near the dorsomedial portions of the brain that were actually driven by greater portions of the cerebral falx being misclassified as grey matter. The grey matter gains detected along the cingulate and central sulci are contaminated with a different error; the smaller CSF spaces at postflight are more likely to be misclassified as grey matter.

### 3.5. Comparison with Manual Segmentation

**Figure 4:**
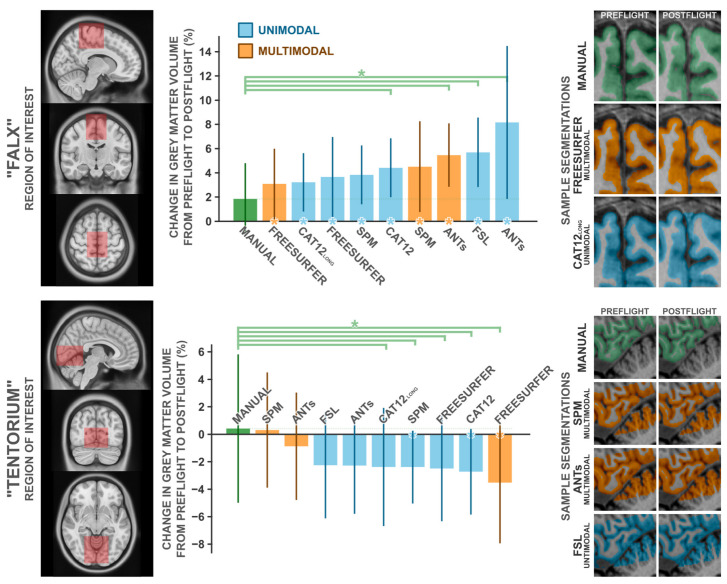
Comparisons of the change in grey matter volume from preflight to postflight between manual segmentation and a handful of commonly employed automated segmentation protocols in the “Falx” ROI (**top panel**) and the “Tentorium” ROI (**bottom panel**). The leftmost panels depict the volume used to quantify grey matter within. Middle panels depict the percent change in grey matter volume detected for each method. Error bars indicate ±1 standard deviation about the group mean. Green asterisks indicate the given method detected significantly (*p*_1*-tailed*_ < 0.05) different grey matter change than that detected from the manual segmentation. Asterisks along the x axis indicate the given method detected a change in grey matter volume that significantly (*p*_1*-tailed*_ < 0.05) deviated from 0. For each ROI, the rightmost panels depict a single subject’s exemplar segmentation from manual segmentation and the best-performing automated segmentations, in that subject’s native space. Note that manual and Freesurfer segmentations do not include the cerebellar grey matter, whereas other packages do. CAT12long refers to the longitudinal segmentation procedure available in CAT12, whereas CAT12 refers to the standard segmentation procedure. *n* = 10.

To quantify the magnitude of these potentially artifactual findings, two experts manually segmented cerebral grey matter partitions from two regions of interest, in ten subjects each, from both preflight and postflight timepoints. We selected these ROIs to capture the two most significant clusters detected in our unimodal voxel-based morphometry analysis, which we depicted in Figure 2 and Figure 3 and believe to be particularly error-prone. The first region, ‘Falx’. is centered about the medial precentral gyrus and cerebral falx, and the second region, ‘Tentorium’, is centered about the ventromedial occipital cortex and cerebellar tentorium. We compared the change in grey matter volume from preflight to postflight as quantified by a handful of commonly used neuroimaging software packages (i.e., SPM, Freesurfer, FSL, ANTs, and CAT12) to those from manual segmentation in both these ROIs in the same set of subjects (Figure 4). In both cases, manual segmentation detected negligible to minimal changes in grey matter volume, where automated procedures overestimated the grey matter increases in the ‘Falx’ ROI and overestimated the losses in the ‘Tentorium’ ROI (with the exception of the ‘Tentorium’ estimations from multimodal implementations in SPM and ANTs).

In the ‘Falx’ ROI (Table A3), manual segmentation identified the most conservative change in grey matter volume with a M (SD) increase of 1.84 (3.12)%. From all assessed automated procedures, the change in grey matter volume computed from Freesurfer’s multimodal segmentation was the most similar to that computed from manual segmentation, at a change of 3.09 (3.07)%; nevertheless, overestimating the change computed from manual segmentation by ~68% (*t*_9_ = 1.032, *p* = 0.329, *d* = 0.326). The remaining packages produced mean estimated grey matter volume changes ranging from 3.22 to 8.16%; overestimating the effects estimated from manual segmentation by ~75 to 343%. Similarly, manual segmentation of the ‘Tentorium’ ROI (Table A4) identified an insignificant increase in grey matter volume, at 0.42 (5.69)%. SPM’s multimodal segmentation identified a similar increase, at 0.31 (4.43)%, whereas all other tested automated packages identified modest [i.e., ANTs’ multimodal pipeline −0.87 (4.15)%] to moderate (all other packages: −2.26 > Δ > −3.52 %) decreases in grey matter volume.

## 4. Discussion

The voxel-based morphometry analyses following unimodal and multimodal segmentation in SPM revealed a strikingly different pattern of spaceflight-related effects. The commonly employed unimodally-segmented analysis (i.e., using T1-weighted MRI images only), identified effects that have considerable overlap with effects previously reported in the literature [6,9,11]. As an example, Koppelmans and colleagues [14] reported widespread grey matter volume decreases in the ventral frontal and temporal lobes, and grey matter volume gains overlapping the precentral gyrus, postcentral gyrus, precuneus, and posterior cingulate; these findings were generally replicated in the present analysis following unimodal segmentation (Figure 1). Koppelmans and colleagues [14]—and others with similar findings [6,9]—cautiously interpreted some of these grey matter changes as indicative of neuroplastic responses to spaceflight, with a specific caveat that findings could be driven by non-neuroplastic processes. Here, we demonstrated that the spaceflight-related effects that we detected using voxel-based morphometry following unimodal segmentation are largely driven by artifacts and are not interpretable as evidence of a neuroplastic response. In fact, the typical tissue losses detected after unimodal segmentation did not appear after the slightly more reliable multimodal segmentation (Figure 2), and the tissue gains that persisted in both analyses were visibly contaminated with large segmentation artifacts (Figure 3). Comparing manual segmentation against automated segmentation procedures in a handful of commonly used software packages revealed that typical usage of automated procedures can produce biased estimates of grey matter volume change (see Figure 4). These findings provide the very first evidence that (a) the typically employed analysis pipelines for detecting volumetric changes are not suitable for investigating astronauts’ neuroplastic changes due to spaceflight, and (b) the use of multimodal segmentation alone does not appear to attenuate these issues sufficiently across the brain such that one can draw valid neuroplastic inferences from these type of analyses.

Collecting large quantities of MRI data from astronauts before and after spaceflight is challenging due to the small number of astronauts flying to space at any given time. Similarly, spaceflight analog studies are often slow and demanding procedures that typically have small sample sizes. As such, it would be ideal if the segmentation errors identified herein could be addressed in previously collected datasets for subsequent re-analysis. Alternate [7] or parameter-optimized tissue classification algorithms may outperform the ‘out of the box’ unimodal and multimodal classification performed by the commonly used software packages (i.e., SPM12, ANTs, CAT12, Freesurfer, and FSL), but any custom algorithm or procedure may need to be specifically tailored to the present problem and population. The simple fact that there is next to no tissue contrast between dura and grey matter on typical T1-weighted images [27] may make accurate distinction between these tissues too challenging for purely automated procedures on T1-weighted images alone. The segmentation errors we presented in Figure 2 and Figure 3 are apparent to the naked eye; therefore, careful manual intervention to an automated segmentation procedure could remove the gross artifactual effects that we have identified.

Manual and semiautomated segmentation procedures are more commonly used in smaller studies of clinical cases, such as those identifying tumors [43,44,45]. Complete manual segmentation of the brain is excessively tedious, but given the relative scarcity of MRI data from astronauts, some degree of manual intervention is justifiable. However, manually removing segmentation errors such that no clearly visible errors remain does not mean that those segmentations are truly error-free. In many regions in the brain, the dura and cortex are touching and have the same intensity on a T1-weighted image, making accurate discrimination between these tissue types extremely difficult, if not impossible, even for experts. Similarly, partial volume estimations [7,46,47] are not typically performed in manual segmentations, leaving the possibility that spaceflight-related fluid shifts will produce consistent errors (i.e., partial voluming) in different parts of the brain that preclude valid neuroplastic interpretations of any prospective results, even after expert intervention following automated segmentation. Irrespective of these challenges, our findings highlight the importance of careful manual quality control in preprocessing and critical evaluation of any given analysis; while the lack of visually apparent errors does not guarantee that a segmentation is accurate, an accurate segmentation will necessarily be free of visually apparent errors. The extraterrestrial environment experienced by astronauts renders invalid many of the implied assumptions present in most default analysis pipelines and procedures that were originally created with different populations and effects in mind. Although we have not provided evidence that the segmentation errors we have identified are also present in spaceflight analog studies utilizing head-down bed rest paradigms, these paradigms are also known to produce salient CSF redistribution within the brain [48,49,50,51] and may be at risk of segmentation errors similar to those identified here. We feel that astronauts, and potentially spaceflight-analog participants, need to be treated as special cases for brain morphometry analysis. Much like other populations where typical procedures are insufficient, this unique population requires unique analyses to meet the validity challenges we have identified.

**Figure A1:**
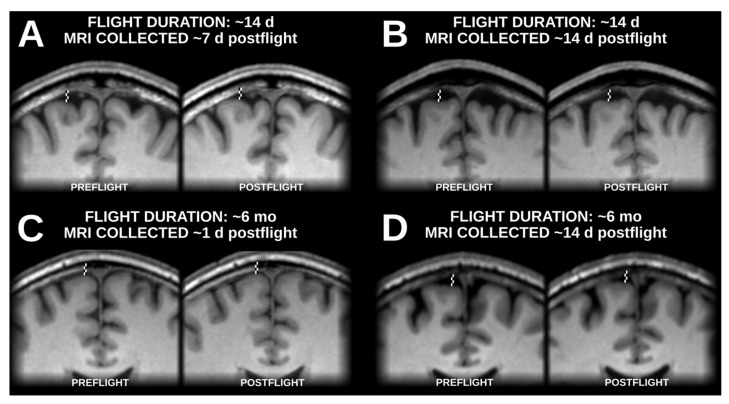
Samples of native-space (i.e., non-normalized) brain positions before and after spaceflight with examples from flights lasting approximately 2 weeks (**A**,**B**) and from flights lasting approximately 6 months (**C**,**D**), with postflight data collection occurring about 2 weeks after landing (**B**,**D**), and significantly closer to landing (**A**,**C**). In all examples, the brain is positioned visibly higher within the skull at postflight time points relative to preflight timepoints.

For future studies of spaceflight, there are a handful of possible solutions that may attenuate the segmentation issues we identified. First, simply delaying the postflight MRI timepoint may allow some fluid shifts and other more direct effects of the spaceflight environment to resolve, leaving neuroplastic effects to be detected. However, this paradigm assumes that the direct effects of spaceflight on the brain are the primary cause of the segmentation errors, which they appear to be, but also assumes that the direct effects return to baseline levels more rapidly than neuroplastic effects of spaceflight, which does not appear to be the case [6,7,12,13]. This also moves against conventional wisdom that one would want to take postflight measurements as soon as possible after astronauts return to earth [52], and may simply not be practical, as differences in fluid distribution are still apparent at postflight delays that approach total mission duration [6,7,13] (also see Figure A1). Delaying postflight data collection also mixes any effects of terrestrial re-adaptation with the effects of spaceflight, which may not simply attenuate the magnitude of spaceflight-related effects, but instead move in a somewhat unique direction of a third, ‘terrestrially-readapted’ neurological state as opposed to simply returning to the ‘spaceflight-naive’ preflight neurological state. Future studies collecting more frequent post-flight neuroimaging data may provide a clearer quantification of the neurological changes during postflight re-adaptation. Such research could reveal an ideal time or times for postflight data collection that would optimize sensitivity to effects of interest and release from bias-inducing effects of no interest.

Secondly, collecting structural MRI data with different protocols that offer better contrast between grey matter and non-neuronal tissue would likely be the best solution to avoid the large segmentation errors confounding our findings. For instance, Diffusion-Weighted Imaging (DWI) utilized by Jillings and colleagues [7], in their investigation of spaceflight-related brain volume changes, may be more robust to some sources of segmentation error typically when segmentation T1-weighted images. The authors utilized a multi-shell DWI sequence and processing pipeline that is far better able to estimate partial volume effects as compared to more typical volumetric procedures, and DWI sequences generally afford a wide variety of other analyses of brain structure (e.g., structural connectivity estimates). However, the authors noted that in their data, some dural structures (i.e., portions of the cerebral falx) have similar diffusional properties as grey matter, allowing for the possibility of mischaracterization of these two tissue types. Additionally, DWI sequences typically offer far lower spatial resolution per unit acquisition time as compared to T1-weighted imaging, a property that offsets some of the benefit of more precise partial volume estimations. On the other hand, some modern structural sequences, such as Multi-echo MPRAGE (MEMPRAGE) and MP2RAGE sequences can produce data that more clearly differentiates grey matter from dura and vasculature [27,53], and appear to produce more reliable (i.e., exhibiting lower test–retest variability) brain volume estimates than the typical MPRAGE sequence used for collecting T1-weighted data [54]. These sequences take slightly longer to acquire than an MPRAGE sequence of equivalent resolution and may not simply be dropped-in as replacements for more traditional MPRAGE images in typical preprocessing pipelines [55]. Utilizing alternate structural imaging modalities [7], selecting the highest-performing extant segmentation approaches or developing novel approaches that perform best with a given imaging modality, and perhaps utilizing higher-resolution acquisitions [56], may additionally resolve artifacts associated with partial voluming errors, such as the small CSF spaces being misclassified as grey matter [55,57] (see Figure 3B). Additional research is needed to identify if segmentations performed on data from alternate structural sequences, such as MP2RAGE, and parameter-optimized or alternate segmentation algorithms are not as prone to the particular errors seen when segmenting traditional MRI images collected in spaceflight or spaceflight analog studies [58,59].

We are not aware of clear evidence that similar validity threats due to segmentation errors are present in other, non-spaceflight research paradigms. However, it is possible that similar errors of varying magnitude are present in other studies in which changes in the rates and location of tissue misclassification can produce artifactual findings. Identifying more reliable data acquisition and processing methods that improve or better leverage tissue contrast, and therefore reduce the reliance on spatial tissue probability priors, may also improve segmentation performance in volumetric analyses of the brain in the presence of atrophy [31], unique morphology [60,61], or normal development [62], in which salient changes in brain and/or CSF volumes may interact with the positioning of the cortex with respect to other tissues and produce a spatial bias in segmentation errors between conditions of interest.

## 5. Conclusions

Spaceflight studies using standard voxel-based morphometry analyses can be contaminated with large segmentation artifacts. These artifacts, likely exacerbated by the direct effects of spaceflight, such as CSF redistribution in the brain, are salient enough that they preclude valid neuroplastic (and therefore cognitive or behavioural) interpretations from grey matter volume change in astronauts. There are a handful of prospective countermeasures that may return validity to these findings, but it is unlikely that a single solution will sufficiently resolve the artifactual findings presented herein. To evaluate any prospective prophylactic measure, the research community will need to identify a reliable paradigm to explicitly quantify the magnitude of the segmentation errors present in different approaches. This could be done by utilizing retrospective data to extensively compare different preprocessing procedures to identify an optimal set of software and parameters that best mimics manual segmentation. Alternatively, prospective research could leverage a hypothetical paradigm in which no neuroplastic changes would be expected, but fluid displacement (and, therefore, brain displacement) would be present. Such a paradigm would allow different analysis pipelines to be tested and optimized to ensure they correctly identify no local brain volume changes without necessitating extensive manual intervention. Once a suitably robust pipeline is identified, it could be employed in spaceflight studies to produce more interpretable findings.

## Data Availability

To protect participant privacy, the data that support these findings are not openly available. Requests for retrospective NASA data should be directed to the NASA Lifetime Surveillance of Astronaut Health Program.

## Appendix A

**Table A1:** Statistically significant (voxel-height two-tailed *p_FWE_* < 0.05) local grey matter volume changes detected using voxel-based morphometry following unimodal segmentation. Clusters sorted in descending order based on peak F statistic. K# indicates the cluster number, K_E_ indicates cluster extent in voxels (1.5 × 1.5 × 1.5 mm), ±indicates the direction of the effect, with positive sign indicating an increase in grey matter volume from preflight to postflight. X, Y, and Z coordinates are reported in MNI space. Labels primarily generated from the Neuromorphometrics atlas provided with SPM12, and the cerebellum atlas provided with FSL.

K#	K_E_	±	X	Y	Z	Cluster Labels
1	2554	−	−2	−74	−6	Lingual cortex, tentorium, peri-occipital CSF
			3	−66	2	Lingual cortex
			−14	−69	−4	Lingual cortex, adjacent white matter
2	2331	+	6	−16	63	Medial precentral gyrus
			−2	−14	58	Supplementary motor area
			−12	−10	62	Supplementary motor area, adjacent white matter
3	969	−	−54	−2	−18	Middle temporal cortex
			−58	−20	−15	Middle temporal cortex
4	247	−	−15	−36	−10	Parahippocampal cortex
5	1390	−	56	−6	−18	Middle temporal cortex
			46	4	−30	Temporal pole, adjacent white matter
			51	4	−22	Superior temporal gyrus
6	184	+	14	−56	54	Precuneus, adjacent white matter
			6	−62	50	Precuneus
7	104	+	−12	−50	−14	Cerebellar lobule V
8	75	−	−33	−3	−38	Inf. temporal & fusiform cortex, adj. white matter
9	451	+	26	−42	50	Superior parietal lobule, adjacent white matter
			24	−34	56	Postcentral gyrus
10	569	−	52	−20	9	Transverse temporal gyrus, planum temporale
			54	3	2	Central operculum, planum polare
11	419	−	−58	−9	6	Planum polare
12	108	−	2	−57	−52	Cerebellar lobule IX
13	204	+	−26	−39	52	Postcentral Gyrus, adjacent white matter
14	155	−	2	36	−20	Medial frontal cortex
15	155	−	−50	−27	12	Planum temporale, parietal operculum

**Table A2:** Statistically significant (voxel-height two-tailed *p_FWE_* < 0.05) local grey matter volume changes detected using voxel-based morphometry following multimodal segmentation. Clusters sorted in descending order based on peak F statistic. K# indicates the cluster number, KE indicates cluster extent in voxels (1.5 × 1.5 × 1.5 mm), ±indicates the direction of the effect, with positive sign indicating an increase in grey matter volume from preflight to postflight. X, Y, and Z coordinates are reported in MNI space. Labels primarily generated from the Neuromorphometrics atlas provided with SPM12, and the cerebellum atlas provided with FSL.

K#	K_E_	±	X	Y	Z	Cluster Labels
1	5254	+	0	−16	56	Supplementary motor area, medial precentral gyrus
			12	−56	56	Precuneus, adjacent white matter
			12	−14	60	Supplementary motor area, adjacent white matter
2	98	+	12	15	54	Supplementary motor area, adjacent white matter
3	79	+	40	−27	50	Postcentral gyrus, adjacent white matter
4	85	+	2	4	46	Supplementary motor area
			9	4	50	Supplementary motor area

**Table A3:** Statistical comparisons between changes in grey matter volume due to spaceflight detected in the ‘Falx’ ROI between segmentation algorithms. UM indicates unimodal, MM indicates multimodal. Statistically significant effects (*α* < 0.05) are emboldened.

		Grey Matter Volume Δ, %			1-Sample *t*-Test against Δ = 0			Paired-Samples *t*-Test against Manual		
Method		*M*	*SD*	*n*	*t*	*p*	*d*	*t*	*p*_1*-tailed*_	*d*
Manual		1.84	3.12	10	1.864	0.095	0.589	-	-	-
SPM	UM	3.83	2.56	10	4.730	**0.001**	1.496	1.722	0.060	0.544
SPM	MM	4.51	3.95	10	3.614	**0.006**	1.143	1.737	0.068	0.549
CAT12	UM	4.42	2.57	10	5.445	**<0.001**	1.722	1.990	**0.039**	0.629
CAT12 long	UM	3.22	2.54	10	4.000	**0.003**	1.265	1.162	0.138	0.367
ANTs	UM	8.16	6.66	10	3.874	**0.004**	1.225	2.830	**0.010**	0.895
ANTs	MM	5.45	2.83	7	5.111	**0.002**	1.932	2.597	**0.020**	0.982
Freesurfer	UM	3.66	3.48	10	3.327	**0.009**	1.052	1.516	0.082	0.479
Freesurfer	MM	3.09	3.07	10	3.182	**0.011**	1.006	1.032	0.165	0.326
FSL	UM	5.69	3.03	10	5.950	**<0.001**	1.881	2.846	**0.010**	0.900

**Table A4:** Statistical comparisons between changes in grey matter volume due to spaceflight detected in the ‘Tentorium’ ROI between segmentation algorithms. UM indicates unimodal, MM indicates multimodal. Statistically significant effects (*α* < 0.05) are emboldened.

		Grey Matter Volume Δ, %			1-Sample *t*-Test against Δ = 0			Paired-Samples *t*-Test against Manual		
Method		*M*	*SD*	*n*	*t*	*p*	*d*	*t*	*p*_1*-tailed*_	*d*
Manual		0.42	5.69	10	0.232	0.822	0.073	-	-	-
SPM	UM	−2.39	2.79	10	−2.704	**0.024**	−0.855	2.267	**0.025**	0.717
SPM	MM	0.31	4.43	10	0.222	0.830	0.070	0.052	0.480	0.016
CAT12	UM	−2.73	3.29	10	−2.624	**0.028**	−0.830	2.519	**0.016**	0.797
CAT12 long	UM	−2.39	4.53	10	−1.663	0.131	−0.526	3.010	**0.007**	0.952
ANTs	UM	−2.28	3.70	10	−1.954	0.082	−0.618	1.485	0.086	0.470
ANTs	MM	−0.87	4.15	9	−0.630	0.546	−0.210	0.606	0.281	0.202
Freesurfer	UM	−2.50	4.05	10	−1.951	0.083	−0.617	1.906	**0.045**	0.603
Freesurfer	MM	−3.52	4.66	10	−2.389	**0.041**	−0.755	2.288	**0.024**	0.723
FSL	UM	−2.26	4.08	10	−1.750	0.114	−0.553	1.803	0.052	0.570

## References

[1] Smith M., Craig D., Herrmann N., Mahoney E., Krezel J., McIntyre N., Goodliff K. The Artemis Program: An Overview of NASA’s Activities to Return Humans to the Moon. Proceedings of the 2020 IEEE Aerospace Conference.

[2] (2021). Open science in space. Nat. Med..

[3] Stahn A.C., Kühn S. (2021). Brains in space: The importance of understanding the impact of long-duration spaceflight on spatial cognition and its neural circuitry. Cogn. Process.

[4] Roy-O’Reilly M., Mulavara A., Williams T. (2021). A review of alterations to the brain during spaceflight and the potential relevance to crew in long-duration space exploration. Npj Microgravity.

[5] Alperin N., Bagci A.M., Lee S.H. (2017). Spaceflight-induced changes in white matter hyperintensity burden in astronauts. Neurology.

[6] Hupfeld K.E., McGregor H.R., Lee J.K., Beltran E.N., Kofman I.S., De Dios E.Y., A Reuter-Lorenz P., Riascos R.F., Pasternak O., Wood S.J. (2020). The Impact of 6 and 12 Months in Space on Human Brain Structure and Intracranial Fluid Shifts. Cereb. Cortex Commun..

[7] Jillings S., Van Ombergen A., Tomilovskaya E., Rumshiskaya A., Litvinova L., Nosikova I., Pechenkova E., Rukavishnikov I., Kozlovskaya I.B., Manko O. (2020). Macro- and microstructural changes in cosmonauts’ brains after long-duration spaceflight. Sci. Adv..

[8] Lee J.K., Koppelmans V., Riascos R., Hasan K.M., Pasternak O., Mulavara A.P., Bloomberg J.J., Seidler R. (2019). Spaceflight-Associated Brain White Matter Microstructural Changes and Intracranial Fluid Redistribution. JAMA Neurol..

[9] Riascos R.F., Kamali A., Hakimelahi R., Mwangi B., Rabiei P., Seidler R.D., Behzad B.B., Keser Z., Kramer L.A., Hasan K.M. (2019). Longitudinal Analysis of Quantitative Brain MRI in Astronauts Following Microgravity Exposure. J. Neuroimaging.

[10] Roberts D.R., Albrecht M.H., Collins H.R., Asemani D., Chatterjee A.R., Spampinato M.V., Zhu X., Chimowitz M.I., Antonucci M.U. (2017). Effects of Spaceflight on Astronaut Brain Structure as Indicated on MRI. N. Engl. J. Med..

[11] Van Ombergen A., Jillings S., Jeurissen B., Tomilovskaya E., Rühl R.M., Rumshiskaya A., Nosikova I., Litvinova L., Annen J., Pechenkova E.V. (2018). Brain Tissue–Volume Changes in Cosmonauts. N. Engl. J. Med..

[12] Van Ombergen A., Jillings S., Jeurissen B., Tomilovskaya E., Rumshiskaya A., Litvinova L., Nosikova I., Pechenkova E., Rukavishnikov I., Manko O. (2019). Brain ventricular volume changes induced by long-duration spaceflight. Proc. Natl. Acad. Sci. USA.

[13] Kramer L.A., Hasan K.M., Stenger M.B., Sargsyan A., Laurie S.S., Otto C., Ploutz-Snyder R.J., Marshall-Goebel K., Riascos R.F., Macias B.R. (2020). Intracranial Effects of Microgravity: A Prospective Longitudinal MRI Study. Radiology.

[14] Koppelmans V., Bloomberg J.J., Mulavara A.P., Seidler R.D. (2016). Brain structural plasticity with spaceflight. Npj Microgravity.

[15] Roberts D.R., Brown T., Nietert P., Eckert M., Inglesby D., Bloomberg J., George M., Asemani D. (2019). Prolonged Microgravity Affects Human Brain Structure and Function. AJNR.

[16] Hupfeld K.E., McGregor H.R., Reuter-Lorenz P.A., Seidler R.D. (2021). Microgravity effects on the human brain and behavior: Dysfunction and adaptive plasticity. Neurosci. Biobehav. Rev..

[17] Lee J.K., Koppelmans V., Pasternak O., Beltran E.N., Kofman I.S., De Dios E.Y., Mulder E.R., Mulavara A.P., Bloomberg J.J., Seidler R.D. (2021). Effects of Spaceflight Stressors on Brain Volume, Microstructure and Intracranial Fluid Distribution. Cerebral Cortex Commun..

[18] Johanson C.E., Duncan J.A., Klinge P.M., Brinker T., Stopa E.G., Silverberg G.D. (2008). Multiplicity of cerebrospinal fluid functions: New challenges in health and disease. Cereb. Fluid Res..

[19] Spector R., Snodgrass S.R., Johanson C.E. (2015). A balanced view of the cerebrospinal fluid composition and functions: Focus on adult humans. Exp. Neurol..

[20] Williams M.A., Malm J. (2019). Mischaracterization of Spaceflight-Associated Neuro-ocular Syndrome. JAMA Neurol..

[21] Maguire E.A., Gadian D.G., Johnsrude I.S., Good C.D., Ashburner J., Frackowiak R.S.J., Frith C.D. (2000). Navigation-related structural change in the hippocampi of taxi drivers. Proc. Natl. Acad. Sci. USA.

[22] Stella A.B., Ajčević M., Furlanis G., Manganotti P. (2020). Neurophysiological adaptations to spaceflight and simulated microgravity. Clin. Neurophysiol..

[23] Marušič U., Meeusen R., Pišot R., Kavcic V. (2014). The brain in micro- and hypergravity: The effects of changing gravity on the brain electrocortical activity. Eur. J. Sport Sci..

[24] Ashburner J., Friston K.J. (2000). Voxel-Based Morphometry—The Methods. NeuroImage.

[25] Ashburner J., Friston K.J. (2005). Unified segmentation. NeuroImage.

[26] Lindig T., Kotikalapudi R., Schweikardt D., Martin P., Bender F., Klose U., Ernemann U., Focke N.K., Bender B. (2018). Evaluation of multimodal segmentation based on 3D T1-, T2- and FLAIR-weighted images—The difficulty of choosing. NeuroImage.

[27] van der Kouwe A.J.W., Benner T., Salat D.H., Fischl B. (2008). Brain morphometry with multiecho MPRAGE. NeuroImage.

[28] Whitwell J.L. (2009). Voxel-Based Morphometry: An Automated Technique for Assessing Structural Changes in the Brain. J. Neurosci..

[29] Ashburner J., Friston K.J. (2001). Why Voxel-Based Morphometry Should Be Used. NeuroImage.

[30] Bookstein F.L. (2001). ‘Voxel-Based Morphometry’ Should Not Be Used with Imperfectly Registered Images. NeuroImage.

[31] Helms G., Kallenberg K., Dechent P. (2006). Contrast-driven approach to intracranial segmentation using a combination of T2- and T1-weighted 3D MRI data sets. J. Magn. Reson. Imaging.

[32] Viviani R., Pracht E.D., Brenner D., Beschoner P., Stingl J.C., Stöcker T. (2017). Multimodal MEMPRAGE, FLAIR, and R2* Segmentation to Resolve Dura and Vessels from Cortical Gray Matter. Front. Neurosci..

[33] Jenkinson M., Beckmann C.F., Behrens T.E.J., Woolrich M.W., Smith S.M. (2012). FSL. NeuroImage.

[34] Friston K.J. (2007). Statistical Parametric Mapping: The Analysis of Functional Brain Images.

[35] Viviani R., Stöcker T., Stingl J.C. (2017). Multimodal FLAIR/MPRAGE segmentation of cerebral cortex and cortical myelin. NeuroImage.

[36] Ashburner J. (2007). A fast diffeomorphic image registration algorithm. NeuroImage.

[37] Sclocco R., Beissner F., Bianciardi M., Polimeni J.R., Napadow V. (2018). Challenges and opportunities for brainstem neuroimaging with ultrahigh field MRI. NeuroImage.

[38] Lorio S., Lutti A., Kherif F., Ruef A., Dukart J., Chowdhury R., Frackowiak R., Ashburner J., Helms G., Weiskopf N. (2014). Disentangling in vivo the effects of iron content and atrophy on the ageing human brain. NeuroImage.

[39] Avants B.B., Tustison N.J., Wu J., Cook P.A., Gee J.C. (2011). An Open Source Multivariate Framework for N-Tissue Segmentation with Evaluation on Public Data. Neuroinform.

[40] Gaser C., Dahnke R., Thompson P.M., Kurth F., Luders E. (2022). Alzheimer’s Disease Neuroimaging Initiative CAT – A Computational Anatomy Toolbox for the Analysis of Structural MRI Data. bioRxiv.

[41] Rai R., Iwanaga J., Shokouhi G., Oskouian R.J., Tubbs R.S. (2018). The Tentorium Cerebelli: A Comprehensive Review Including Its Anatomy, Embryology, and Surgical Techniques. Cureus.

[42] Glasser M.F., Goyal M.S., Preuss T.M., Raichle M.E., Van Essen D.C. (2014). Trends and properties of human cerebral cortex: Correlations with cortical myelin content. NeuroImage.

[43] McGrath H., Li P., Dorent R., Bradford R., Saeed S., Bisdas S., Ourselin S., Shapey J., Vercauteren T. (2020). Manual segmentation versus semi-automated segmentation for quantifying vestibular schwannoma volume on MRI. Int. J. CARS.

[44] Wilke M., de Haan B., Juenger H., Karnath H.-O. (2011). Manual, semi-automated, and automated delineation of chronic brain lesions: A comparison of methods. NeuroImage.

[45] Işın A., Direkoğlu C., Şah M. (2016). Review of MRI-based Brain Tumor Image Segmentation Using Deep Learning Methods. Procedia Comput. Sci..

[46] Khademi A., Venetsanopoulos A., Moody A.R. (2014). Generalized method for partial volume estimation and tissue segmentation in cerebral magnetic resonance images. J. Med. Imaging.

[47] Tohka J. (2014). Partial volume effect modeling for segmentation and tissue classification of brain magnetic resonance images: A review. WJR.

[48] Koppelmans V., Pasternak O., Bloomberg J.J., De Dios Y.E., Wood S.J., Riascos R., Reuter-Lorenz P.A., Kofman I.S., Mulavara A.P., Seidler R.D. (2017). Intracranial Fluid Redistribution But No White Matter Microstructural Changes During a Spaceflight Analog. Sci. Rep..

[49] Koppelmans V., Bloomberg J.J., De Dios Y.E., Wood S.J., Reuter-Lorenz P.A., Kofman I.S., Riascos R., Mulavara A.P., Seidler R.D. (2017). Brain plasticity and sensorimotor deterioration as a function of 70 days head down tilt bed rest. PLoS ONE.

[50] Li K., Guo X., Jin Z., Ouyang X., Zeng Y., Feng J., Wang Y., Yao L., Ma L. (2015). Effect of Simulated Microgravity on Human Brain Gray Matter and White Matter—Evidence from MRI. PLoS ONE.

[51] Roberts D.R., Zhu X., Tabesh A., Duffy E.W., Ramsey D.A., Brown T.R. (2015). Structural Brain Changes following Long-Term 6° Head-Down Tilt Bed Rest as an Analog for Spaceflight. AJNR Am. J. Neuroradiol..

[52] Van Ombergen A., Demertzi A., Tomilovskaya E., Jeurissen B., Sijbers J., Kozlovskaya I.B., Parizel P.M., Van de Heyning P.H., Sunaert S., Laureys S. (2017). The effect of spaceflight and microgravity on the human brain. J. Neurol..

[53] Marques J.P., Kober T., Krueger G. (2010). MP2RAGE, a self bias-field corrected sequence for improved segmentation and T1-mapping at high field. Neuroimage.

[54] Yan S., Qian T., Maréchal B., Kober T., Zhang X., Zhu J., Lei J., Li M., Jin Z. (2020). Test-retest variability of brain morphometry analysis: An investigation of sequence and coil effects. Ann. Transl. Med..

[55] Duché Q., Saint-Jalmes H., Acosta O., Raniga P., Bourgeat P., Doré V., Egan G.F., Salvado O. (2017). Partial volume model for brain MRI scan using MP2RAGE. Hum. Brain Mapp..

[56] Zaretskaya N., Fischl B., Reuter M., Renvall V., Polimeni J.R. (2018). Advantages of cortical surface reconstruction using submillimeter 7 T MEMPRAGE. NeuroImage.

[57] Akkus Z., Galimzianova A., Hoogi A., Rubin D.L., Erickson B.J. (2017). Deep Learning for Brain MRI Segmentation: State of the Art and Future Directions. J. Digit Imaging.

[58] Fujimoto K., Polimeni J.R., van der Kouwe A.J., Reuter M., Kober T., Benner T., Fischl B., Wald L.L. (2014). Quantitative comparison of cortical surface reconstructions from MP2RAGE and multi-echo MPRAGE data at 3 and 7T. NeuroImage.

[59] Singh M.K., Singh K.K. (2021). A Review of Publicly Available Automatic Brain Segmentation Methodologies, Machine Learning Models, Recent Advancements, and Their Comparison. Ann. Neurosci..

[60] Gau K., Schmidt C.S.M., Urbach H., Zentner J., Schulze-Bonhage A., Kaller C.P., Foit N.A. (2020). Accuracy and practical aspects of semi- and fully automatic segmentation methods for resected brain areas. Neuroradiology.

[61] King D.J., Novak J., Shephard A.J., Beare R., Anderson V.A., Wood A.G. (2020). Lesion Induced Error on Automated Measures of Brain Volume: Data From a Pediatric Traumatic Brain Injury Cohort. Front. Neurosci..

[62] Peterson M.R., Cherukuri V., Paulson J.N., Ssentongo P., Kulkarni A.V., Warf B.C., Monga V., Schiff S.J. (2021). Normal childhood brain growth and a universal sex and anthropomorphic relationship to cerebrospinal fluid. J. Neurosurg. Pediatr..

